# Effects of post-lithography cleaning on the yield and performance of CVD graphene-based devices

**DOI:** 10.3762/bjnano.10.34

**Published:** 2019-02-05

**Authors:** Eduardo Nery Duarte de Araujo, Thiago Alonso Stephan Lacerda de Sousa, Luciano de Moura Guimarães, Flavio Plentz

**Affiliations:** 1 Departamento de Física, CCE, Universidade Federal de Viçosa, Viçosa, Minas Gerais, 36570-900, Brasil; 2 Departamento de Física, ICEx, Universidade Federal de Minas Gerais, C.P. 702, Belo Horizonte, Minas Gerais 30123-970, Brasil

**Keywords:** CVD graphene, defects, mobility, well-ordered domain

## Abstract

The large-scale production of high-quality and clean graphene devices, aiming at technological applications, has been a great challenge over the last decade. This is due to the high affinity of graphene with polymers that are usually applied in standard lithography processes and that, inevitably, modify the electrical proprieties of graphene. By Raman spectroscopy and electrical-transport investigations, we correlate the room-temperature carrier mobility of graphene devices with the size of well-ordered domains in graphene. In addition, we show that the size of these well-ordered domains is highly influenced by post-photolithography cleaning processes. Finally, we show that by using poly(dimethylglutarimide) (PMGI) as a protection layer, the production yield of CVD graphene devices is enhanced. Conversely, their electrical properties are deteriorated as compared with devices fabricated by conventional production methods.

## Introduction

The unique properties of graphene, such as high conductivity, high carrier mobility at room temperature, high sensitivity of the electrical properties to surface phenomena and the existence of several routes for its surface functionalization, grant this 2D material plenty of application possibilities [1–8]. Among the several synthesis methods of high-quality graphene, chemical vapor deposition (CVD) stands out as one of the most promising methods for large-scale production [9]. However, the challenges in mass-production of graphene-based devices are a great obstacle for the full development of an electronic industry based on graphene [10]. This is due to the difficulty to avoid structural degradation and chemical contamination of graphene in lithography processes [11]. Because of this, in the present work we investigate by Raman spectroscopy and electrical transport measurements the effects of different post-photolithography cleaning methods on the yield and performance of CVD-based graphene devices.

## Experimental

**Figure 1:**
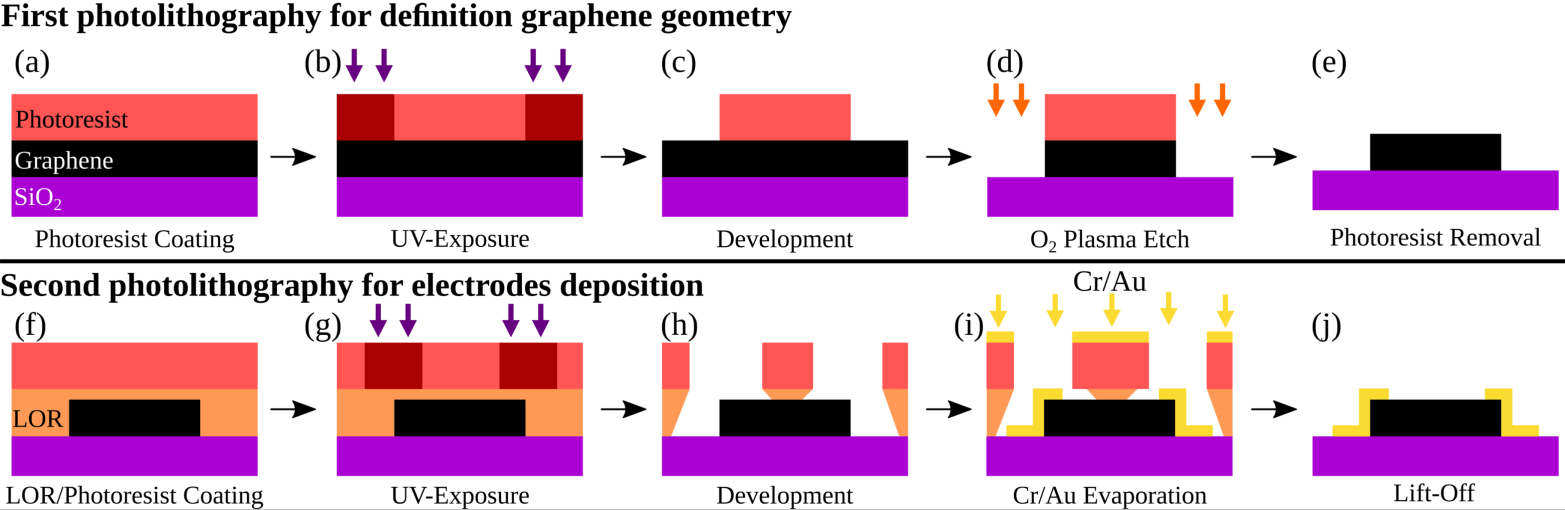
First photolithography step for the definition of the graphene structure: a) photoresist coating onto graphene; b) exposure of photoresist using direct laser writing; c) development of exposed areas; d) etching of graphene in O_2_ plasma; e) photoresist removal. Second photolithography step for electrode fabrication: f) LOR/photoresist coating onto graphene; g) exposure of photoresist using direct laser writing; h) development of exposed areas; i) thermal evaporation of Cr/Au; j) lift-off process.

We made use of CVD graphene on top of a 300 nm thick SiO_2_ layer, which was purchased from Graphene Platform. The graphene devices were produced in the field-effect transistor configuration (GFET) in two photolithography steps (Figure 1). The first step was employed for defining the graphene device geometry, the second step was used for the fabrication of the electrodes.

In the first photolithography step, graphene was coated with a 1340 nm thick layer of photoresist (AZ 1512 HS of MicroChemicals.com) (Figure 1a). Then, the geometry of the device was defined by direct laser writing photolithography (Figure 1b), followed by the development of the exposed photoresist in AZ 351B (1:4) developer (MicroChemicals.com, Figure 1c). After this development, the excess of graphene was removed with O_2_ plasma (Figure 1d). Finally, we removed the photoresist layer (Figure 1e) using different protocols, as we describe next. In the second photolithography step, the previously patterned graphene was coated with a 820 nm thick layer of poly(dimethylglutarimide) (PMGI)-based resist, LOR5A from Microchem.com, followed by 1340 nm thick layer of photoresist AZ 1512 HS (Figure 1f). After the UV exposition, the development of the exposed photoresist produces undercut profiles, seen in Figure 1g,h. Then, 5/100 nm of Cr/Au were thermally evaporated on the samples and the lift-off procedure (Figure 1j) was performed according to procedure “P1” or procedure “P2”.

**Figure 2:**
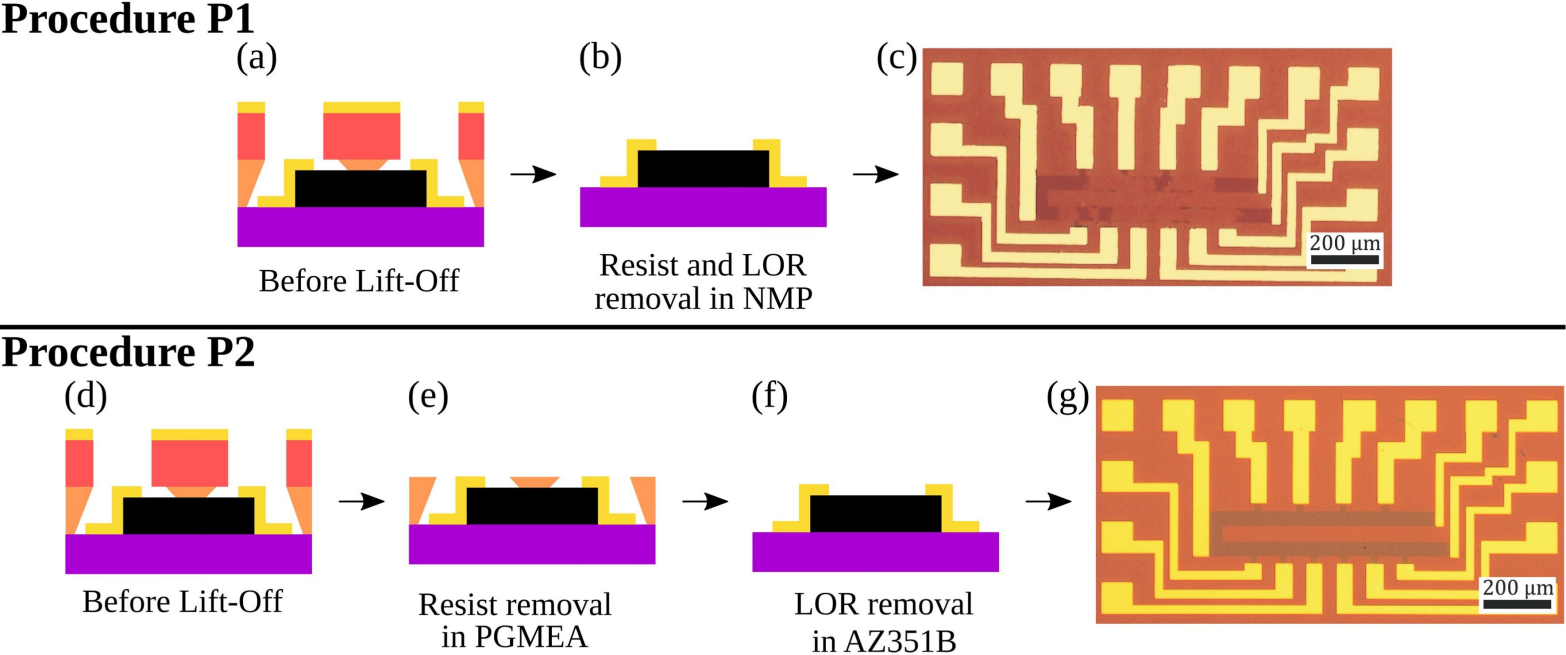
Procedures P1 and P2 for photoresist removal and lift-off. Procedure P1: (a, b) The LOR/resist layer was removed in an unique step, using NMP, followed by rinsing in IPA and DI. c) Graphene device after lift-off; Procedure P2: d) The resist layer was removed first using 1-methoxy-2-propanol acetate (PGMEA), then the graphene device was washed out in IPA and DI. e) and f) Next, the LOR layer was removed in AZ351B, followed by rinsing in IPA and DI. g) Devices with continuous graphene were obtained by procedure P2. The scale bars shown in c) and g) measure 200 μm.

In procedure called P1, the steps of photoresist removal (Figure 1e) and the lift-off as well (Figure 1j) were performed whith the resist stripper, *N*-methyl-2-pyrrolidone (NMP), followed by rinsing in isopropanol (IPA) and deionized water (DI, Figure 2a,b). Figure 2c shows that this procedure seriously damages graphene. We attribute this to the well-known high solubility of graphene in NMP, which tends to promote the delamination of the graphene layer from the substrate. Since procedure P1 is quite destructive, its yield of high-quality GFETs was 10% in a total of 60 devices.

In order to improve the efficiency of device production, we removed the photoresist and LOR in a step-by-step manner, using a second procedure, called P2. At first, the photoresist was removed by 1-methoxy-2-propanol acetate (PGMEA), a solvent towards which LOR is inert (Figure 2d,e). Then, the remaining LOR layer that covered the graphene film, was removed in AZ351B (1:4) (Figure 2f). Procedure P2 was found to be less aggressive than procedure P1 and it increased the efficiency of device production up to 85%, in a total of 60 devices (Figure 2g). In fact, high-yields have been reported in the literature when PMGI is applied as support scaffold in the transfer of CVD graphene [12–13].

We performed Raman spectroscopy and electrical transport measurements, at room temperature, to correlate the size of well-ordered domains in graphene with its carrier mobility. The Raman spectroscopy was performed using an InVia Renishaw Raman spectrometer with a 514.5 nm laser and the electrical transport measurements were performed using a lock-in amplifier (SR-830 Stanford Research Systems).

## Results and Discussion

**Figure 3:**
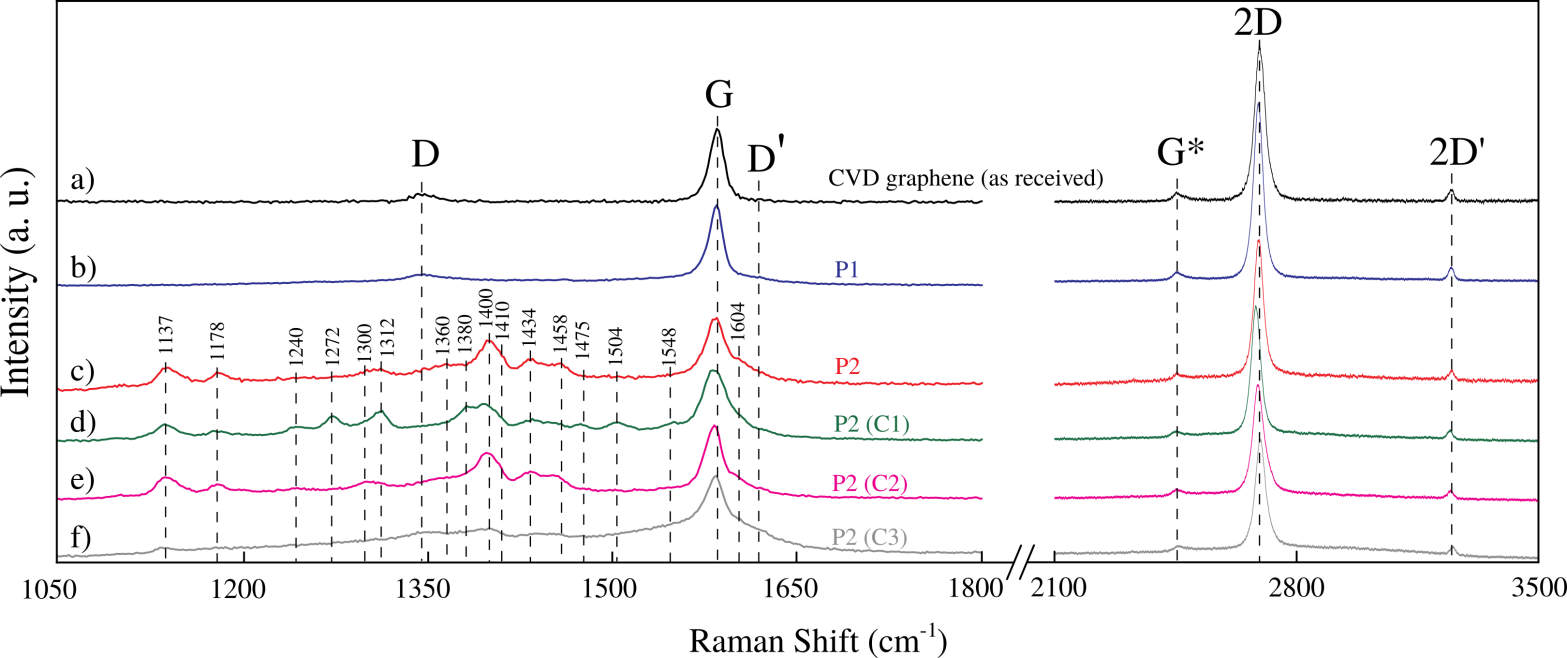
Raman spectra of CVD graphene devices (from top to bottom): graphene as-received; graphene device fabricated by procedure P1 (immediately after lift-off); graphene device fabricated by procedure P2 (immediately after lift-off); graphene device fabricated by procedure P2 and after cleaning with NMP (C1); graphene device fabricated by procedure P2 and after cleaning with DMF (C2); and graphene device fabricated by procedure P2 and after cleaning with H_2_/Ar annealing (C3).

Figure 3a shows the Raman spectrum of the as-received CVD graphene (as a reference), while Figure 3b and Figure 3c show, respectively, the Raman spectra of the graphene devices produced by procedures P1 and P2. The Raman spectrum of the graphene processed by procedure P1 (Figure 3b) is very similar to the Raman spectrum of the reference CVD graphene (Figure 3a). It indicates that the lift-off using NMP leaves just a negligible amount of chemical residues on graphene. However, Figure 3c indicates that the PMGI polymer is not completely removed in the lift-off process carried out by procedure P2. This can be asserted by the fact that the bands at 1137, 1178, 1300, 1312, 1400, 1410, 1434, 1458 and 1604 cm^−1^, shown in Figure 3c, are associated to PMGI polymer [12,14–16]. Thus, the huge increase of the device production using procedure P2 is accompanied by a significant contamination with PMGI during lift-off.

To remove residual PMGI from the graphene devices produced by procedure P2, we attempted three different cleaning methods. The first method (C1) consisted of cleaning the devices in a NMP bath. In the second cleaning method (C2), we used *N,N*-dimethylformamide (DMF) to clean the graphene devices. Finally, a third method (C3) consisted of graphene cleaning by H_2_/Ar (1:1) annealing at 300 °C for 2 h [17–18]. Nonetheless, we have observed that both cleaning methods C1 and C2, which respectively employ NMP and DMF, were not capable of removing the residual PMGI polymer from the graphene surface. It is evidenced by the fact that the Raman spectrum of graphene device fabricated by procedure P2 (Figure 3c), and the Raman spectra of both graphene devices produced by procedure P2 and cleaned with NMP (Figure 3d) and DMF (Figure 3e) present the same features associated to the PMGI polymer. Regarding the H_2_/Ar annealing, Figure 3f shows that this process did not remove the residual PMGI polymer completely from the graphene surface, because some Raman bands associated with the polymer are still present.

Structural disorder in graphene can be quantified through the *I*_D_/*I*_G_ intensity ratio. For laser excitation with wavelength λ_L_, the characteristic size of well-ordered domains in graphene can be estimated as:

[1]
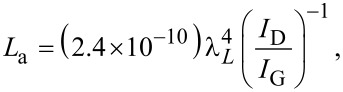


which properly applies to *L*_a_ *>* 4 nm [19–21].

**Figure 4:**
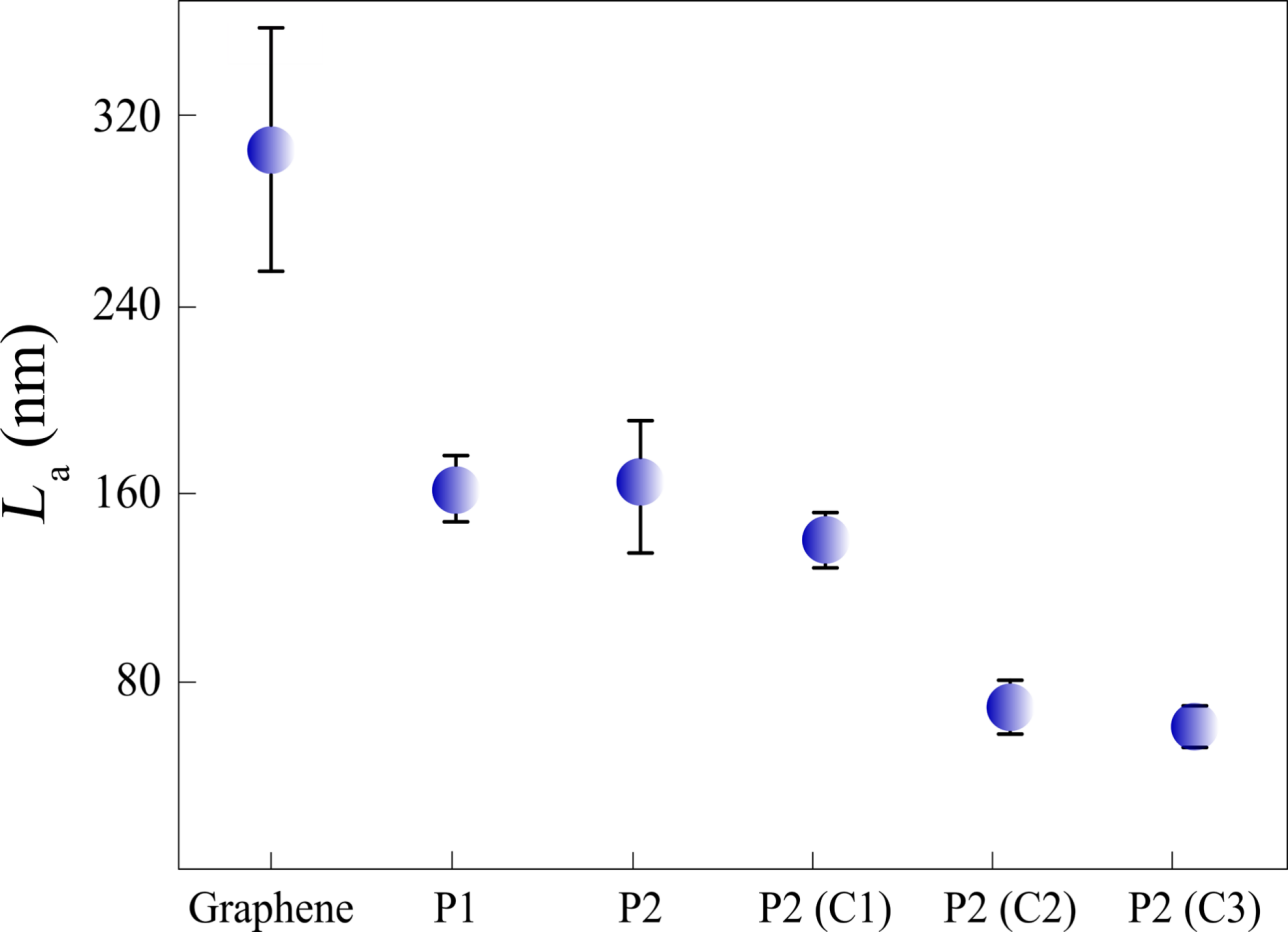
Size of well-ordered domains (*L*_a_) for CVD graphene (as-received), for graphene devices fabricated by procedures P1 and P2, and for graphene devices fabricated by procedure P2 and further cleaned by NMP (C1), DMF (C2), H_2_/Ar annealing (C3).

According to Figure 4, the size of well-ordered domains for as-received CVD graphene is *L*_a_ = 307 ± 50 nm. The size of well-ordered domains for graphene processed by both procedures P1 and P2 decreases to *L*_a_ ≈ 160 nm. From this, we infer that these procedures introduce approximately the same quantity of structural defects in graphene. Furthermore, the value of *L*_a_ showed no expressive decrease as the devices fabricated by procedure P2 are cleaned by method C1. However, when the cleaning is performed by methods C2 and C3, the size of well-ordered domains shows a further decrease, which suggests an increase of structural disorder in graphene [17,22–23].

**Figure 5:**
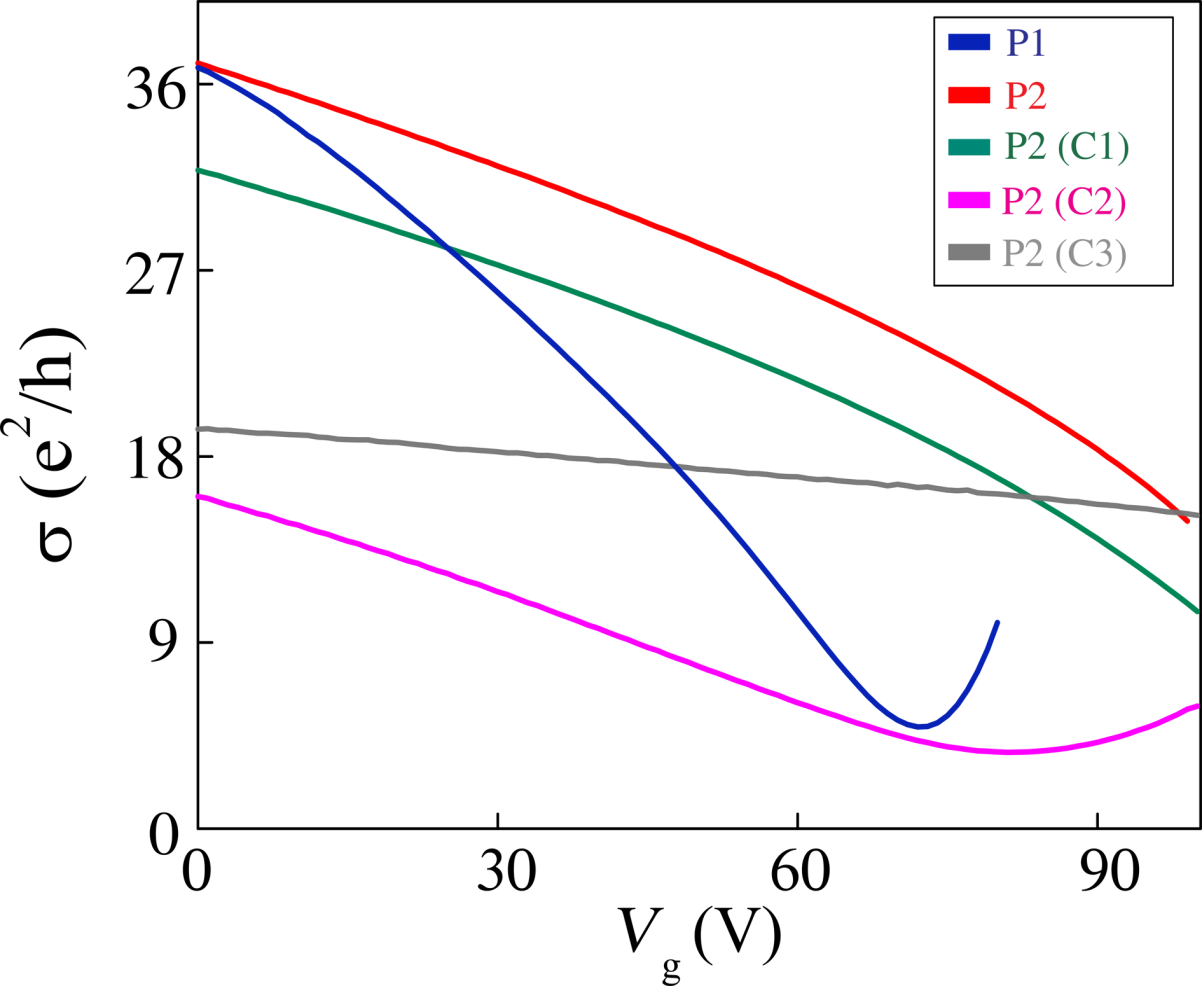
Conductivity σ as a function of the gate voltage *V*_g_ for graphene devices fabricated by procedures P1 and P2, and for graphene devices produced by procedure P2 and then cleaned by C1, C2 and C3 methods.

Therefore, we have a situation in which the cleaning methods either do not clean the graphene surface or promote some degree of cleaning, but at the expense of introducing defects. In order to correlate the results obtained from Raman spectroscopy with the electrical transport properties of the graphene devices, we performed conductivity measurements as a function of gate voltage (*V*_g_) for several devices produced by different lift-off procedures and cleaning methods. Since the neutrality point was beyond 100 V for several devices, we only consider here the transport by holes. Figure 5 shows that graphene conductivity, σ, behaves as a sublinear function of the gate voltage, *V*_g_. This sublinear behavior is associated to a weak-point disorder in graphene, which emerges as a carrier density independent residual resistivity, ρ_s_. The strong disorder and the charged-impurity disorder are responsible for the resistivity (μ*ne*)^−1^, in which μ is the mobility and *n* is the carrier density [8,21].

Since *n* = α*V*_g_, where α = 7.2 × 10^10^ cm^−2^, we were able to find the resistivity ρ_s_ that linearizes the relation

[2]
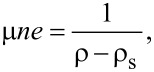


where ρ = 1/σ is the measured resistivity of graphene [24]. Then, the graphene mobility was obtained by a linear fit of (*rho* − ρ_s_)^−1^ as a function of *V*_g_.

**Figure 6:**
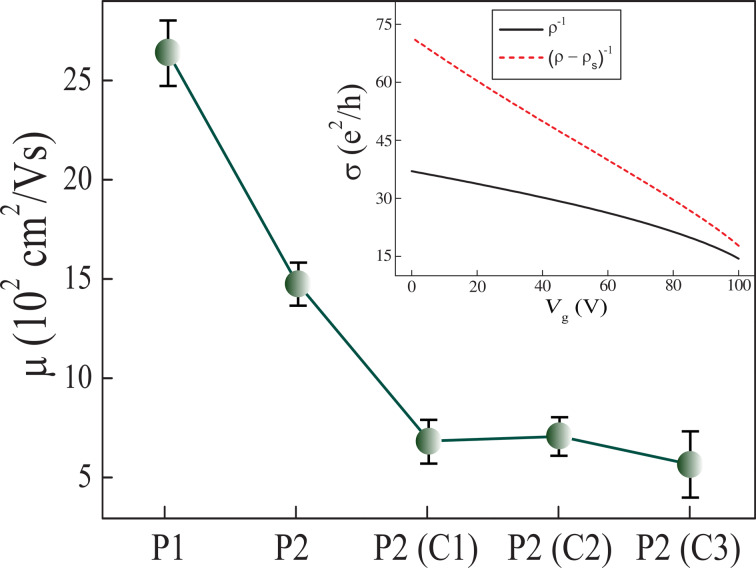
Hole-mobility values of graphene devices fabricated by different procedures, P1 and P2, and by different cleaning methods, C1, C2 and C3. Inset: graphene conductivity ρ^−1^ and (ρ − ρ_s_)^−1^ as a function of *V*_g_ for a representative device produced by procedure P2.

Figure 6 shows the hole-mobility values of graphene devices produced by different lift-off procedures, P1 and P2, and by different cleaning methods, C1, C2 and C3. The inset in Figure 6 shows as example ρ^−1^ and (ρ − ρ_s_)^−1^ as a function of *V*_g_ for a graphene device produced by procedure P2. The mobility of the graphene devices produced by procedure P1, P2, C1, C2 and C3 follows a different trend than the size of the well-ordered domains in graphene. For example, according to Figure 4, the graphene devices produced by procedures P1 and P2 have nearly the same size of well-ordered domains. However, the mobility of the graphene devices produced by procedure P1, μ = (2.6 ± 0.2) × 10^3^ cm^2^/Vs, is considerably larger than the mobility of the graphene devices produced by procedure P2, which is μ = (1.5 ± 0.1) × 10^3^ cm^2^/Vs. This result strongly suggests that the size of well-ordered domains in graphene is not the unique mechanism that limits its mobility in this case.

Within the semiclassical Boltzmann transport formalism, the total mobility can be described in terms of the mobility μ_d_, related to crystal lattice defects, and in terms of the mobility μ_c_, associated to charged impurities scattering centers, according to [24–26]:

[3]
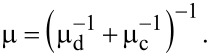


The lattice defects can be modeled as a deep potential well of radius *R*, which gives rise to the mobility μ_d_, as:

[4]
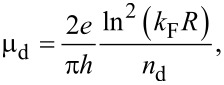


where *n*_d_ *~* (2*RL*_a_)^−1^ is the defect density and *k*_F_ corresponds to the Fermi wave vector [21,24]. Using the Thomas–Fermi screening approach, the mobility related to charged impurities can be written as:

[5]
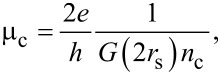


where *G*(2*r*_s_) is an analytical function that takes into account the interaction strength in graphene, *r*_s_ is the interaction parameter and *n*_c_ is the density of charged impurities [24–26].

As the graphene devices produced by procedures P1 and P2 have approximately the same size of well-ordered domains (Figure 4), we assume that these devices have nearly the same density of lattice defects. Therefore, according to Equation 4, the mobility related to lattice defects, μ_d_, is the same for these devices. Thus, charged impurities must be the scattering source that distinguishes the mobility of the graphene devices produced by procedures P1 and P2. Figure 3b shows that the graphene devices produced by procedure P1 have just a negligible extent of residual of PMGI on their surface, while Figure 3c shows a considerable amount of residual PMGI on the surface of P2 graphene. So, we believe that PMGI molecules adsorbed on the graphene surface act as scattering source of charged impurities. Hence, as the density of charged impurities in P2 graphene devices is larger than in P1 graphene devices, the mobility related to charged impurities (Equation 5) is greater for the latter than for the former. Consequently, according to Equation 3, the total mobility, μ, is larger for P1 graphene devices, which is quantitatively consistent with Figure 6.

Additionally, Figure 6 also shows that the mobility of the graphene devices produced by procedure P2 and cleaned by method C1 (NMP) is substantially lower than the mobility of the graphene devices produced by procedure P2 with no further cleaning. According to Figure 3c,d and to Figure 4, P2 and C1 graphene devices hold roughly the same extent of residual PMGI and have practically the same size of well-ordered domains. So, in this case, we believe that NMP just spreads out PMGI molecules along the graphene surface. Thus, it may lead to a more uniform distribution of charged impurities over the graphene surface that, in turn, decreases even more the mobility of the C1 graphene devices. Finally, Raman characterization shows that DMF (Figure 4e) and H_2_/Ar annealing (Figure 4f) are not efficient in removing PMGI molecules from the graphene surface. Otherwise, these cleaning methods introduce lattice defects in graphene, which increase the value of *n*_d_. Therefore, the considerable density of lattice defects, *n*_d_, and the density of charged impurities, *n*_c_, are responsible for the low mobility of C2 and C3 graphene devices.

## Conclusion

We performed electrical transport measurements and Raman spectroscopy investigations to compare the proprieties of CVD graphene-based devices processed using conventional photolithography with devices produced by a recently developed method using LOR as a sacrificial layer. We found that the PMGI molecules introduce disorder in graphene, which impairs the performance of the CVD-graphene based devices. We then applied the most common methods of post-photolithography cleaning in order to remove PMGI molecules. We were able to correlate the electrical mobility to the well-ordered domain size in devices that went through different cleaning procedures. We conclude that the use of LOR as sacrificial layer improves the CVD graphene device production yield, but impair the overall electronic performance of the devices.
